# Precision nutrition: Maintaining scientific integrity while realizing market potential

**DOI:** 10.3389/fnut.2022.979665

**Published:** 2022-09-02

**Authors:** Silvia Berciano, Juliana Figueiredo, Tristin D. Brisbois, Susan Alford, Katie Koecher, Sara Eckhouse, Roberto Ciati, Martin Kussmann, Jose M. Ordovas, Katie Stebbins, Jeffrey B. Blumberg

**Affiliations:** ^1^Friedman School of Nutrition Science and Policy, Tufts University, Boston, MA, United States; ^2^Advanced Personalization Ideation Center, PepsiCo Inc., Purchase, New York, NY, United States; ^3^Novo Nordisk Inc., Plainsboro Township, NJ, United States; ^4^Bell Institute of Health and Nutrition, General Mills, Inc., Minneapolis, MN, United States; ^5^FoodShot Global, New York, NY, United States; ^6^Barilla G&R, Parma, Italy; ^7^German Entrepreneurship, Cambridge, MA, United States; ^8^Nutrition and Genomics Laboratory, JM-USDA-Human Nutrition Research Center on Aging, Tufts University, Boston, MA, United States

**Keywords:** precision nutrition, personalized nutrition, omics, genetics, microbiome, metabolic health, wearable devices

## Abstract

Precision Nutrition (PN) is an approach to developing comprehensive and dynamic nutritional recommendations based on individual variables, including genetics, microbiome, metabolic profile, health status, physical activity, dietary pattern, food environment as well as socioeconomic and psychosocial characteristics. PN can help answer the question “What should I eat to be healthy?”, recognizing that what is healthful for one individual may not be the same for another, and understanding that health and responses to diet change over time. The growth of the PN market has been driven by increasing consumer interest in individualized products and services coupled with advances in technology, analytics, and omic sciences. However, important concerns are evident regarding the adequacy of scientific substantiation supporting claims for current products and services. An additional limitation to accessing PN is the current cost of diagnostic tests and wearable devices. Despite these challenges, PN holds great promise as a tool to improve healthspan and reduce healthcare costs. Accelerating advancement in PN will require: (a) investment in multidisciplinary collaborations to enable the development of user-friendly tools applying technological advances in omics, sensors, artificial intelligence, big data management, and analytics; (b) engagement of healthcare professionals and payers to support equitable and broader adoption of PN as medicine shifts toward preventive and personalized approaches; and (c) system-wide collaboration between stakeholders to advocate for continued support for evidence-based PN, develop a regulatory framework to maintain consumer trust and engagement, and allow PN to reach its full potential.

## Introduction

Nutrition is a fundamental pillar of health, and diet is the modifiable factor that exerts the greatest impact on human health and wellbeing (1). Dietary recommendations have traditionally been based on a one-size-fits-all approach which assumes that individual nutritional requirements and responses mimic the average response observed in study populations (2). The advancement of personalized nutrition or precision nutrition (PN) strategies has improved our understanding of how factors such as genetic, microbiome, and metabolic signatures, may predict whether what we eat supports or harms our health and to what degree (3).

Studies in the field of nutritional genomics have unveiled associations between genetic factors and metabolic responses to food, nutrient requirements, dietary preferences, and disease outcomes (4–7). Advances in this and other areas of PN have added new dimensions that help explain the variability in responses observed in otherwise well-controlled trials of diet and nutrients (8). In particular, promising research results support the predictive potential of assessments of the gut microbiome and metabolome—among other factors—and showcase the individual but malleable qualities of our biology (9, 10).

As we bring new perspectives to the multiple dimensions of food and health, we are also overcoming some of the barriers created by previous reductionistic thinking about nutrition. It is in this context that PN is driving the scientific journey toward a more personalized, predictive, and integrative systems approach to understanding how nature and nurture interact to shape our health and wellbeing.

## The precision nutrition approach: One size does not fit all

**Figure 1:**
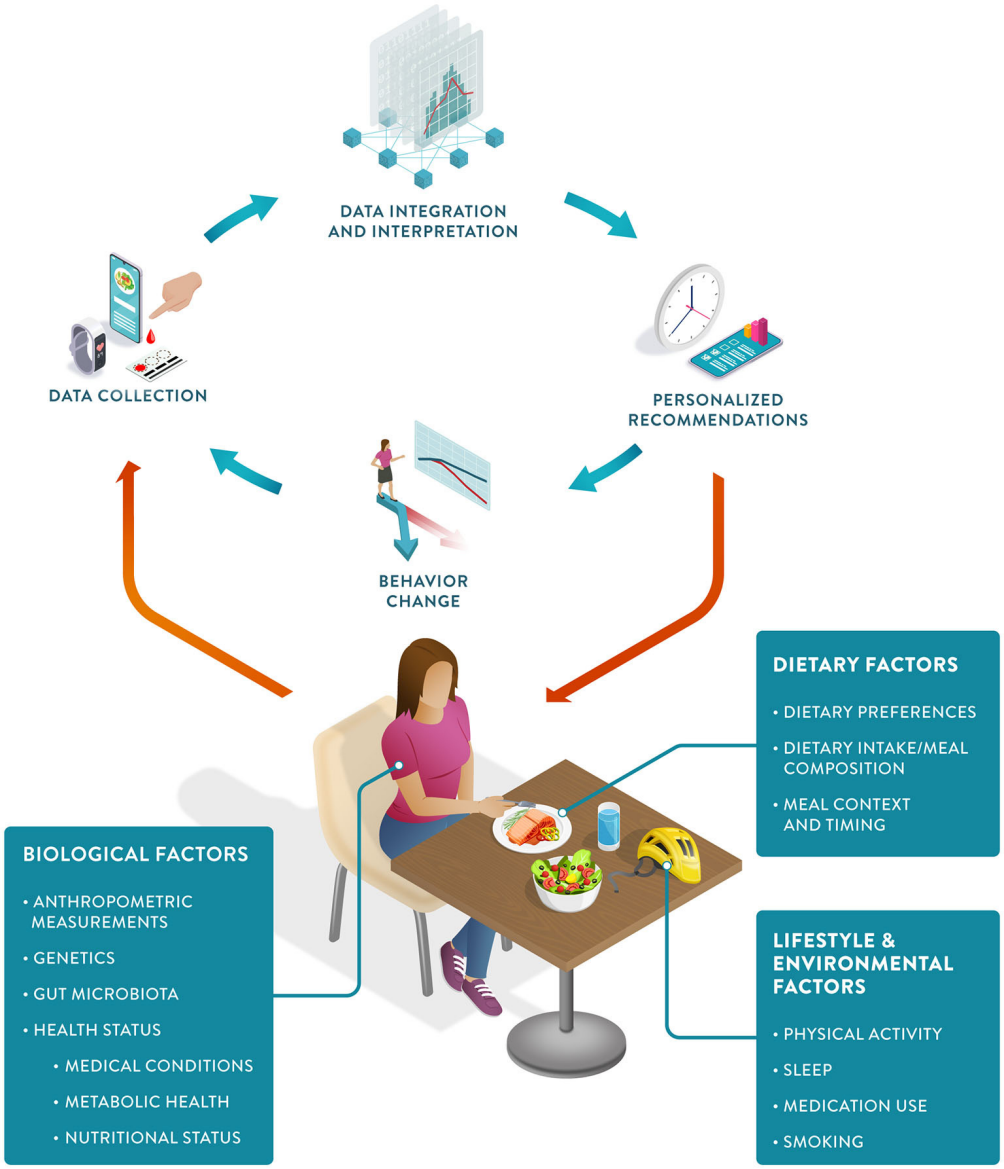
The Precision Nutrition approach: The PN feedback loop starts with individual data collection, including biological, dietary, and other lifestyle factors. Data integration and interpretation enable personalized recommendations, which in turn can induce positive behavioral change, ultimately resulting in improved health outcomes.

PN can be defined as an approach that uses individual data to predict how a person will respond to specific foods or dietary patterns and tailors dietary recommendations to their individual needs. These personalized recommendations are expected to elicit behavioral changes that would lead to improvements in the health trajectory of the person (Figure 1).

PN represents an advancement from both traditional dietary advice and earlier personalized approaches (11). Despite recognized causal links between diet and health outcomes, traditional dietary intervention strategies to reduce the burden of chronic diseases have had a limited impact (5). This is due to a combination of poor adherence to dietary recommendations and different individual responses to the same food and dose (12–14). PN has the potential to tackle both challenges. Personalized recommendations have been shown to increase compliance, and predictions of the direction and magnitude of an individual's response to food allow for the development of more effective recommendations tailored to their specific needs and metabolism (15).

Aided by a wealth of data now gathered *via* wearable devices, smartphones, and diagnostic tests, PN holds the promise of a more cost-effective approach to health promotion (16, 17). These data can be analyzed and integrated using computational methods to generate both qualitative and quantitative “just-in-time” nutritional recommendations for the individual client or patient.

As PN continues to advance, it has the potential to further enhance our understanding and application of nutrition and significantly impact outcomes such as health maintenance, resilience and restoration, fertility, physical capacity, and cognitive performance. Key attributes of the PN approach are listed in Table 1.

**Table 1:** Key attributes of precision nutrition.

**P**ersonalized	Individual data is collected and used as input
**R**eliable	Derived recommendations are linked to improved outcomes
**E**vidence-based	Supported by scientific evidence and robust methodology
**C**omplex	Specialized knowledge and tools are key to its development
**I**ntegrative	Intra- and inter-individual variability depends on multiple predictors
**S**ystematic	Data is collected, analyzed, and presented systematically
**E**volving	Dynamic recommendations evolve as the individual changes and/or additional data become available

**Figure 2:**
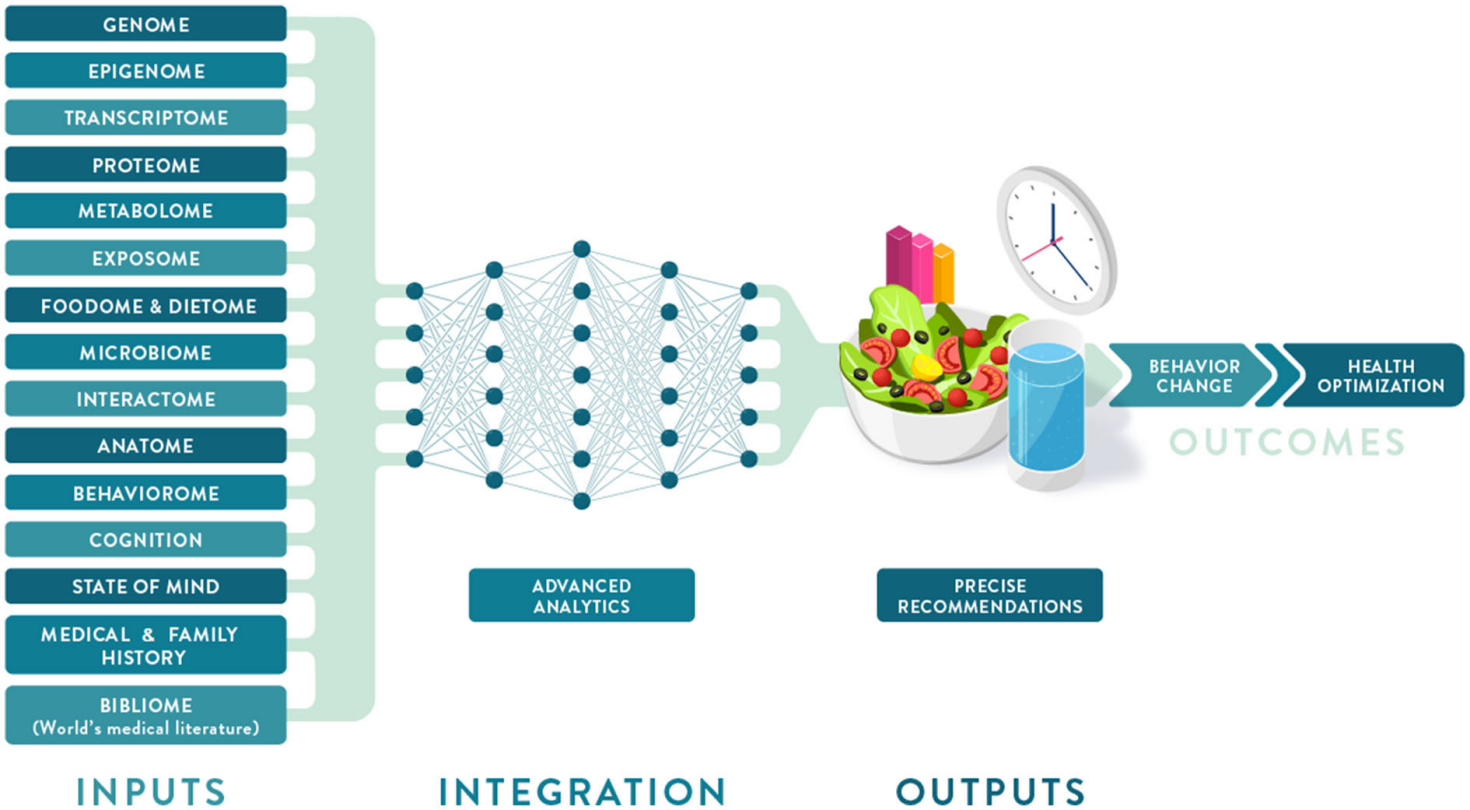
Deep phenotyping and multiomic integration in Precision Nutrition. Multiple data layers that make up an individual deep phenotyping profile are integrated and analyzed using a neural network approach to provide optimized dietary recommendations leading to behavior change and improved health outcomes.

Individual variation in dietary behaviors and responses has been shown to be multi-faceted and includes genetics, metabolic profiling, and meta-omic signatures (such as metagenomics and meta-transcriptomics) as well as psychological, anthropometric, sociodemographic, and environmental factors (Figure 2). To condense some of these layers of information into more manageable inputs, many of these predictors are presented as “-types” or signatures. While “genotype” generally refers to a single locus in our genome, metabotypes, nutritypes, ageotypes, and phenotypes refer to composite measures that combine a large number of variables related to our metabolism, diet, aging, and other traits.

It is clear that the foundation of PN has been built largely on evidence from omic studies that are more focused on molecular biology and nutritional biochemistry than environmental and social drivers of eating behavior. However, considering additional characteristics, including sensorial responses, personal circumstances, values, attitudes, behaviors, and social determinants of health (SDOH), will facilitate the development of PN solutions that are adequately tailored to, accepted, and adopted by the individual, resulting in improved lifestyles and lasting health.

## Big data and analytics in precision nutrition

Advances in data acquisition and analytics have enabled omics to build connections between large data sets to further understand the multiple facets of food intake behavior and nutrient metabolism. Technological advances in omic sciences, artificial intelligence (AI), and sensors (especially wearable devices) also have the potential to revolutionize how nutrition research is conducted and how dietary insights are presented to and used by the public.

Traditional dietary assessment methods, including food frequency questionnaires, diet records, and recalls, have limited resolution to provide a precise intake profile and can be burdensome to complete. The development of mobile apps offering image recognition to quantify meals and wearable sensors to detect and capture nutrient intake, along with barcode scanners to facilitate the recognition of packaged foods, may result in more precise, real-time, and user-friendly dietary assessments (18).

The growing presence of sensors and personal electronic devices in homes will complement and increase the accuracy of image- and questionnaire-based methods to track dietary intake. Next-generation wearables will be able to continuously and non-invasively monitor blood glucose and other biomarkers before “lab-on-a-chip” implants are created, which will combine sensing capabilities with delivery systems. Smart appliances and toilets will collect data on food intake, nutrient status, dietary responses, and health biomarkers of each consenting member of the household (19). Smart pills are already considered an inexpensive tool for direct sampling of microbial communities in the gastrointestinal tract. They can provide new insights into the role of diet in mediating host-microbe interactions and metabolism (20, 21). At-home sampling and testing using fecal collection kits, dried blood spot cards, and continuous glucose monitors are now commercially available. More comprehensive devices under development may replace some of the current options and enable higher-resolution and real-time nutrient, behavior and health tracking.

Multiomic profiling plays a major role in research directed at identifying sets of biomarkers relevant to health maintenance and disease prevention. While the advent of ultra-connected devices and the Internet of Things can revolutionize nutrition and health data collection, AI has already changed how big data are analyzed and interpreted (22). AI is instrumental in the analysis of massive real-world data collected using wearables or diagnostic tools to better detect patterns and predict health trajectories. Common applications of machine learning algorithms in nutrition include the discovery and validation of new bioactive ingredients, integration of dietary and health data, development of predictive models and recommendations to optimize health outcomes.

PN tools available to consumers (many of whom are early adopters of this technology, eager to share their insights and data) are becoming more numerous and accessible, fueling interest in new study designs in personal and citizen science. These approaches may reduce the time an individual spends on an intervention before a positive or adverse event is detected (23). Importantly, trials directed to PN can reveal subclinical departures from health to disease, thereby enabling the discovery of early markers of deviation from a healthy trajectory that inform disease prevention and health maintenance (24).

## Developing personalized nutrition products and services

The translation of PN science into products and services can be enhanced by considering the balance of benefits and risks for both consumers and patients. Benefits may include the improvement of a specific health outcome, the convenience of user-friendly digital tools, and the efficacy of a more personalized approach. On the other hand, risks may result from the high cost of repeated omic testing, the time burden for users due to complex programs, and unmet expectations where gaps exist between the science and product claims. Risks also include concerns regarding trust, privacy, and control of data.

Several privately and publicly funded large-scale studies are underway to gather key data and develop the necessary knowledge and methods to elucidate which metrics are most important, what degree of granularity or resolution is necessary, and which signatures of health and disease should receive priority for testing (3, 25). These findings are expected to promote innovation with validated and novel PN products and services in the healthcare and food industries. In addition, open-source tools are being developed to support individuals and their healthcare providers, offering more personalized dietary and lifestyle recommendations to complement population-based recommendations like the Dietary Guidelines for Americans (DGA) (2).

Some issues of concern regarding the development of commercial PN products and services include insufficient scientific evidence supporting effectiveness, the limited predictive power of the underlying algorithms, and unsubstantiated claims. The majority of available commercial PN products and programs are collecting data and refining algorithms as they are being used. This progressive generation of data and knowledge could be at the expense of the consumer if the interpretations or recommendations being generated are incorrect or ineffective, e.g., when they suggest causality without evidence. Even when there are statistically significant associations between specific factors and health-related outcomes, several commercial solutions appear to lack validation, sufficient predictive power, clinical relevance, and/or actionable advice. The level of phenotyping and comprehensive predictive analytics found in some advanced research settings do not seem feasible for direct commercial applications at this time.

The PN market is largely unregulated and dominated by small companies. Even wellbeing solutions that are generally considered to pose little threat to the individual carry a risk of misleading consumers if such products or services are not rigorously designed and their benefits clearly evaluated and communicated. The growing demand for PN tests, personalized diets, and supplements in the absence of a reasonable regulatory framework could lead to an erosion of consumer trust. The development and adoption of evidence-based PN solutions ultimately depend on consumers sharing personal data, so credibility, transparency, and trust are essential to the responsible growth of this industry.

Future efforts in PN are expected to involve the use of key biomarker panels and integrated analyses. Separate layers of data can be collected by different partners, but agreed-upon standards are needed to facilitate merging datasets so that a more accurate interpretation can be achieved. PN solutions can also be tailored to well-defined consumer or patient groups which encompass individuals with similar needs. Scientific rigor, relevance of diet/health predictors, convenience, and access are essential considerations in the development and democratization of PN. Making these attributes a priority will accelerate innovation in PN and its integration into healthcare strategies for individualized disease prevention, treatment, and care.

## Precision nutrition and the future of healthcare

Human life expectancy has increased by three decades over the last century. However, this lifespan extension has not been matched by an improvement in healthspan, i.e., years lived without disease (26). Suboptimal diets are responsible for 1 in 5 adult deaths globally (27). The epidemic of diet-related chronic conditions accounts for 90% of the $3.5 trillion in annual health care expenditures in the U.S. (28).

However, the impact of poor diets on human health has not been fully recognized in healthcare. Public health approaches have addressed this burden from either the perspective of hunger and undernutrition (including food and nutrition insecurity) or that of overweight and obesity (focused on promotion of dietary quality and reduction of energy density) (29). Metabolic health is a current metric of interest and defined based on a combination of risk factors: fasting glucose (<100 mg/dL), hemoglobin A1c (<5.7%), blood pressure (systolic <120 and diastolic <80 mmHg), triglycerides (<150 mg/dL), high-density lipoprotein cholesterol (≥40/50 mg/dL for men/women), anthropometrics (waist circumference <102/88 cm for men/women), and not taking any related medication (30). Only 12% of adults in the US are metabolically healthy, including less than one-third of normal-weight adults (27). PN can facilitate metabolic health assessment by using more sensitive markers, enabling earlier detection of metabolic dysregulation and providing more effective dietary strategies to regain metabolic health.

Deep phenotyping can provide a better understanding of individual risk factors, dietary responses, and metabolic regulation. Paired with early detection and correction of health trajectories, deep phenotyping holds a promise for increasing our healthspan. Digital twins are high-resolution models of a deeply phenotyped subject that can be computationally subjected to unlimited different nutritional interventions (31). Though the cost and complexity of digital twins in precision medicine have been compared to the Human Genome Project, digital twin approaches may be useful in simulating individual dietary effects and generating recommendations for health optimization (32). This concept represents a very high level of personalization in contrast to current approaches that rely on stratification to more simply distinguish or cluster individuals with similar needs or responses. Unlocking the full potential of such big data approaches exceeds the capability of existing computers. However, it may be feasible in 5–10 years when hybrid machines that combine quantum and conventional processing become essential tools in the analysis of highly complex datasets involving multiple interacting variables.

Individualized or n-of-1 trials are one-person trials where the number of measured variables and the sampling frequency can capture the intra-individual variability across a health trajectory, enabling comparisons between different interventions and empirical determination of optimal diet for a specific person (33). Although such findings are not expected to be generalizable, they are compatible with the goal of clinical practice—the care of individual patients (23). The n-of-1 study design represents a promising type of PN research, particularly when multiple n-of-1 studies are conducted in a coordinated manner and can be aggregated to identify subgroup effects. Aggregated coordinated n-of-1 studies cluster individuals with similar characteristics or responses (e.g., responders vs. non-responders), can help achieve greater resolution in nutrition science and expand the research approaches used to substantiate PN programs. Therefore, they present excellent opportunities to advance this field.

Non-invasive wearable- and app-enhanced measurements of health and lifestyle parameters such as blood pressure, blood glucose, body composition, dietary intake, and physical activity are key enablers of consumer empowerment. Their proactive use in healthcare can promote a shift to measurable prevention and healthier habits compared to the current reactive approach, in which healthcare is sought primarily when health declines. The “Quantified Self” trend refers to wearable-based and app-enabled self-measurements of health parameters by end-users, consumers, and patients (34). This is a key driver for the adoption of innovative PN solutions, as consumers can autonomously measure the benefits of dietary and lifestyle changes. However, the real value is created when these personal data are put into context: apps and tools need to be linked to credible databases to generate sound, actionable, and personalized advice.

Early adopters of PN are generally individuals driving their own health journey, often without expert advice or financial incentives. They tend to be younger, healthier, more affluent, and possess higher nutrition and health literacy (35). While this group can benefit from PN programs, connecting consumers and patients whose need for PN assessment and recommendations is more urgent should be a priority. Healthcare systems can be instrumental in fostering broader adoption of PN by emphasizing its role in health promotion and disease prevention and shifting its principal focus from a disease-centric model. This approach will require the education of patients and healthcare providers, many of whom are not knowledgeable about how diet can drive health outcomes (36).

**Figure 3:**
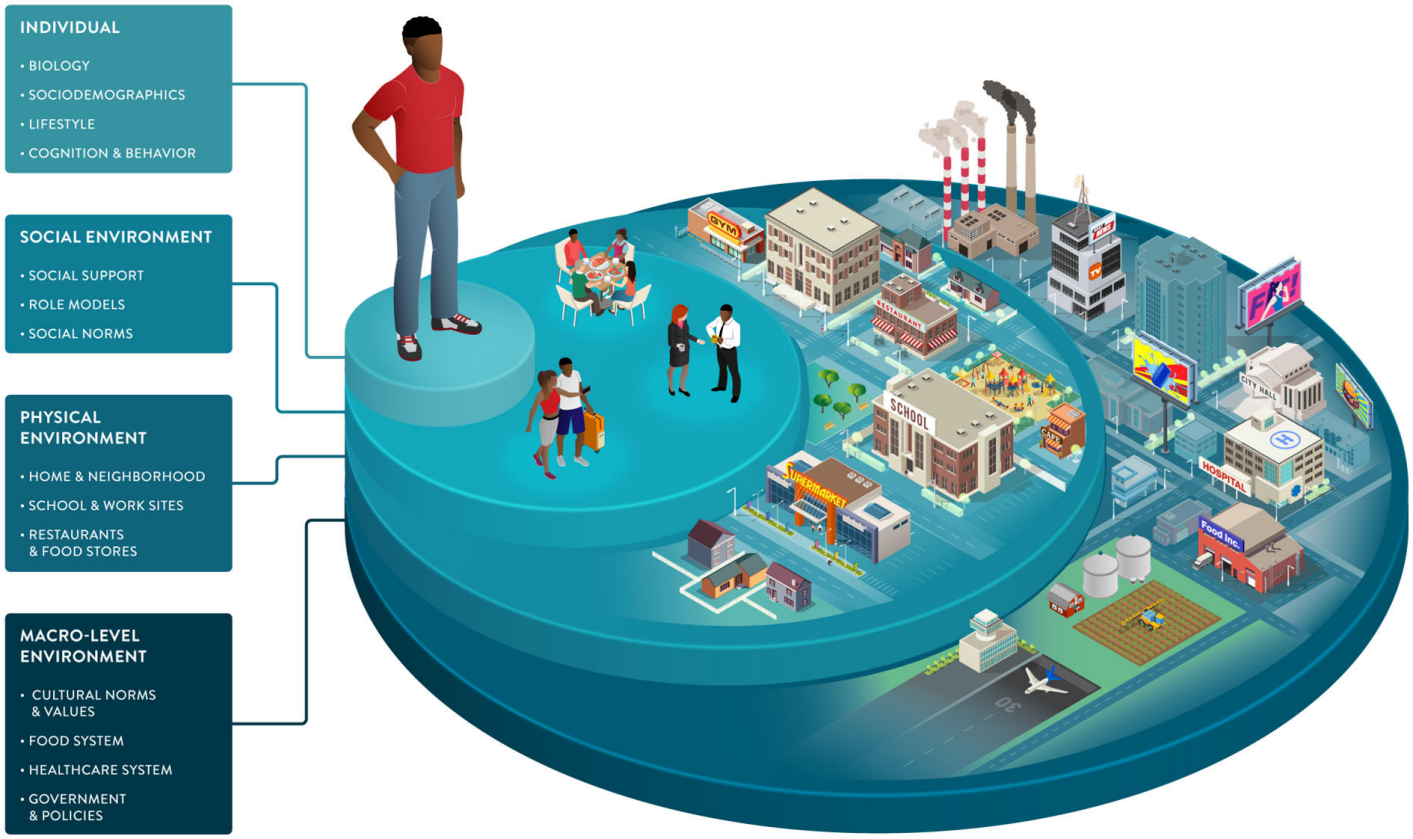
Socio-ecological framework highlighting individual, social, and environmental dimensions that can drive dietary behaviors and responses. Sociodemographic characteristics and the environments in which we live influence our food choices and interact with many of the individual factors impacting our health and wellbeing. The framework illustrates the multiple dimensions that can be considered to understand dietary choices better and enable healthy behavior change tailored to individuals and their environments.

Environmental factors and SDOH impact food choices and the opportunities and barriers that support or hinder healthy behaviors. As part of the broader environment, macro-level factors play an indirect but powerful role in driving individual health. This dimension includes healthcare systems, public health policies, and food systems, from harvesting and processing to commercialization and marketing (Figure 3).

Advancing PN requires the development of precise measures of exposures, behaviors, and susceptibilities in diverse populations. Shifting the focus from treatment to prevention and delivering the right intervention to the right population at the right time can be achieved with Precision Public Health approaches that complement individual-focused Precision Nutrition strategies (37).

This path to integrated healthcare is not short: moving from reactive treatment to proactive prevention approaches will require time, investment, training, and clear guidelines but is expected to generate significant value. In a report from the McKinsey Company (38), it is estimated that the effective use of big data by the U.S. healthcare sector would create $300 billion in value every year. Most of this estimated value would be derived from the identification of the most clinically effective and cost-effective treatments based on data already being generated by health care providers.

Insurers will also play a crucial role in PN adoption, providing incentives when clients engage with PN measures. Healthcare systems can reduce costs when pharmacological treatments are replaced by or combined with less expensive nutritional solutions, especially for chronic and lifestyle-related conditions that cannot be sustainably managed by pharmaceutical means alone (39). However, PN approaches must first show evidence of clinical efficacy and cost-effectiveness. Robust returns on investment in the form of improvements in clinical outcomes, patient and provider experience, and lower healthcare costs would justify reimbursement. In addition to healthcare systems, PN could also serve as an integral part of workplace wellness programs, encouraging employees to be proactive about health maintenance and deploying evidence-based health apps that provide actionable lifestyle recommendations (40).

## Best practices and standards in precision nutrition

Nutrition regulators will need to develop policies regarding evidence generation, approval, and reimbursement of PN solutions. This applies to approaches delivered in healthcare settings as well as direct-to-consumer products and services. Given the fast pace of research and innovation in this field, it is critical for academia, industry, and policymakers to work together and generate effective regulatory frameworks and guidelines, starting with industry standards and best practices.

The need for and growing interest in PN across different organizations and industries calls for a joint effort to establish a consensus framework covering definitions, science, commercialization, and communication. Innovation will move faster and more effectively if a common language between academia, industry, and regulatory bodies is established. Standards are the basis for mutual understanding of PN products and services, and for improving transparency and acceptance of this approach as a strategy in healthcare and a solution for consumers. Moreover, best practices in PN science, commercialization, and communication are key to enabling the replication and comparison of findings and fostering trust and credibility with consumers, patients, and healthcare providers.

Standards will also apply to novel study designs and analytical approaches which are necessary to overcome experimental limitations and establish effective PN science. Although the n-of-1 approach is not novel, it has gained traction over the last decade (41). Such designs require additional methodological and statistical considerations to ensure proper data analysis and interpretation to generate sound personalized advice (42).

Currently, the lack of diversity in PN research, as in many research areas, is a limitation as it could lead to inappropriate application of PN algorithms and impede equitable implementation. The National Institutes of Health (NIH) Precision Nutrition initiative addresses this issue by leveraging the All of Us cohort. Such diverse and inclusive populations are crucial to minimizing information gaps in PN trials, from ethnicity to environmental exposures to varied SDOH. Closing this gap with an emphasis on underrepresented populations will help make effective and personalized dietary approaches possible for all in the near future (35).

Developing and implementing research best practices is key to conducting high-quality science and generating the robust evidence foundation needed to substantiate PN products, services, and solutions. This is important as most medical and nutrition research is funded by industry, not by public sources (43). PN solutions should provide evidence of specific health benefits or amelioration of health symptoms or pathology. Rising consumer demand drives PN companies to constantly reassess their markets and innovate rapidly, launching new products in a field that does not yet have a strong evidence foundation. Caution is warranted in putting marketing ahead of science. In moving forward to establish PN practices, there are also legal challenges such as ensuring privacy and ethical use of data when collecting and processing personal information (44).

Currently, there is no specific framework for evaluating PN solutions. Potential regulatory approaches include federal regulation and industry self-regulation. While it is clear that industry standards and federal policies can complement each other, strengths and limitations specific to each regulatory approach are listed in Table 2. Academics, industry leaders, and policymakers must work together to establish scientific standards to define which claims should be allowed and how to substantiate them. That set of common rules, in turn, must be translated into regulatory frameworks.

**Table 2:** Strengths and limitations of industry self-regulation and government regulation of precision nutrition.

**Industry self-regulation**	**Government regulation**
**Strengths:** •More rapid implementation •Better market understanding with consumer insights •Pushing boundaries of innovation and entrepreneurship	**Strengths:** •Perceived as more credible and directed to consumer protection •Greater potential to level the playing field
**Limitations:** •Perceived as self-serving •Lack of consensus or compliance between companies regarding standards and practices	**Limitations:** •Slow to develop consensus and lags behind consumer needs •Perceived as overreaching •May limit innovation with excessive regulation

While government regulation and enforcement guidelines for PN appear inevitable, they will need to be prioritized and funded and will take years to create. A proactive consortium of stakeholders may help establish a consensus framework for the definition, science, practice, and communication of PN before developing a broader regulatory framework.

## Advocacy and communication strategies for precision nutrition

Effective communication of PN is key to promoting trust and the adoption of evidence-based solutions. The novelty and inherent complexity of PN, combined with the legacy of nutrition controversies and misinformation, contribute to the confusion that consumers, healthcare providers, and regulators face when encountering, interpreting, and formulating nutrition messages.

Growing access to information continues to transform the way people think about nutrition and its connections to health. There are several channels through which the general public can access nutrition information; some reflect general recommendations (e.g., DGA), while others provide a biased view that creates opportunities for nutrition misinformation (45). The lack of nutrition and health literacy is a major challenge, and nutrition communications outside of trustworthy sources are fragmented, especially on social media (46).

**Figure 4:**
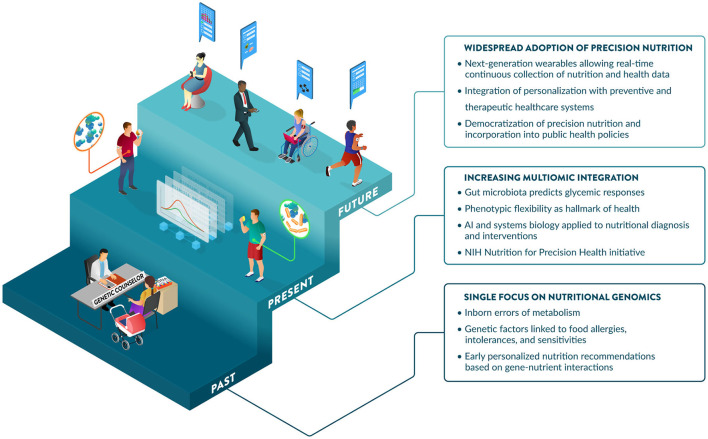
The past, present, and future of Precision Nutrition. The origins of PN are linked to the discovery of inborn errors of metabolism and later more prevalent gene-diet interactions affecting nutrient metabolism and requirements. The contributions of nutritional genomics to PN are complemented by additional omic and input layers, providing increased predictive power of diet-related outcomes, including glycemic responses. In addition to traditional markers measured in the fasting state, insights from dynamic postprandial responses strengthen the importance of phenotypic flexibility, which is now considered a hallmark of health. Large-scale initiatives from NIH and elsewhere are refining systems biology approaches and AI-driven tools to integrate data layers, predict outcomes, and derive effective dietary recommendations tailored to the biology, environment, and specific needs of individuals. The future may offer increased opportunities for the adoption of PN, enabled by wearables that provide a user-friendly and seamless collection of dietary and health data, which will be leveraged by healthcare to lower costs and improve population health, aided by public health policies.

Communications regarding the science of PN often portray different degrees of excitement about this field: from overly optimistic to openly obstructive or visibly skeptical (47). Support from the NIH has significantly increased the profile of the PN field. This includes the NIH Nutrition for Precision Health Initiative (48), which has a strong focus on PN in their 2020–2030 strategic plan (25), and a notable investment of $170 million for a new program to develop algorithms to predict individual responses to food and dietary routines (49). PN is maturing and expected to play a major role in disease prevention and health promotion over the next decade. Further, perceptions of benefits, costs, risks, and uncertainties associated with PN influence consumer attitudes and acceptance (50). Promoting nutrition and health literacy and developing best practices and standards for the effective communication of PN to different audiences will be critical to enabling its adoption. Guidance from professional societies and government agencies on responsible communications regarding the application of PN today and its promise for the future would help address this challenge. Significant advances and investments in large PN studies are generating the data, methods, and knowledge to enable the incorporation of PN into healthcare strategies as early as 2030 (48). Key developments and hallmarks of the early days, present and foreseeable future of PN are illustrated in Figure 4.

## Conclusions and recommendations

PN is a data-driven approach to assessing health that tailors dietary recommendations to individual needs. When fully embedded in the healthcare system, PN should have an impact on both personal and public health. Advancing the science and the adoption of PN will require a significant investment in multidisciplinary collaborations that translate the fast-moving technological advances in omics, sensors, AI, and big data management and analytics into powerful and user-friendly tools. To allow PN to reach its full potential while maintaining consumer trust and engagement, regulatory frameworks from the industry and/or government will need to be established along with guidance on relevant ethical and legal aspects of the practice. Healthcare professionals and providers will need to be trained to provide PN services, supporting a broad and equitable adoption of PN. Collaboration between stakeholders in the world of nutrition and health will be required to expand the scientific and clinical evidence of the efficacy and effectiveness of PN and ensure that system-wide barriers to access and coverage can be overcome.

## Author contributions

KS and SB conceived the idea for this perspective. SA, TB, RC, SE, KK, MK, JO, and JB provided input to inform the review outline and draft. SB and JF drafted the manuscript. All authors contributed to reviewing, editing, and approving the final version of the manuscript.

## Funding

This work was supported by the Tufts Food & Nutrition Innovation Council (FNIC). The FNIC Precision Nutrition Working Group is a collaboration between the Food & Nutrition Innovation Institute in the Friedman School of Nutrition Science and Policy at Tufts University and members of its Council, a multi-stakeholder coalition working to rethink the global food system with a focus on health, equity and sustainability.

## Conflict of interest

Author TB is employed by PepsiCo Inc. and co-chair of the FNIC Precision Nutrition Working Group. Author SA is an employee of Novo Nordisk Inc. and is a stockholder and a co-chair of the FNIC Precision Nutrition Working Group. Author KK is an employee of General Mills, Inc. Author SE is an employee of FoodShot Global. Author RC is an employee of Barilla G&R. Author MK is affiliated with German Entrepreneurship, USA. Author JO serves on scientific advisory boards for Nutrigenomix, Zoe Global, GNC, PepsiCo, and Weight Watchers. Author JB serves on scientific advisory boards for Segterra, Inc. (Inside Tracker) and January.ai, Inc. (outside of the submitted work). The remaining authors declare that the work was conducted in the absence of any commercial or financial relationships that could be construed as a potential conflict of interest.

## Publisher's note

All claims expressed in this article are solely those of the authors and do not necessarily represent those of their affiliated organizations, or those of the publisher, the editors and the reviewers. Any product that may be evaluated in this article, or claim that may be made by its manufacturer, is not guaranteed or endorsed by the publisher.

## Abbreviations

AI: artificial intelligence
DGA: Dietary Guidelines for Americans
NIH: National Institutes of Health
PN: precision nutrition
SDOH: social determinants of health
U.S.: United States.
